# FORTY WINKS: PILOT MULTISITE PRAGMATIC CLINICAL TRIAL TO IMPROVE SLEEP FOR NURSING HOME RESIDENTS WITH DEMENTIA

**DOI:** 10.1093/geroni/igad104.2652

**Published:** 2023-12-21

**Authors:** Kate Smith, A Lynn Snow, Robert Morgan, Megan McCullough, Rosa Baier, Ellen McCreedy, Kathy Richards, Christine Hartmann

**Affiliations:** University of Alabama, Tuscaloosa, Alabama, United States; University of Alabama, Tuscaloosa, Alabama, United States; University Of Texas Health Science Center, Houston, Texas, United States; University of Massachusetts Lowell, Lowell, Massachusetts, United States; Brown University School of Public Health, Providence, Rhode Island, United States; Brown University School of Public Health, Providence, Rhode Island, United States; University of Texas at Austin, Austin, Texas, United States; VA Bedford Healthcare System, Bedford, Massachusetts, United States

## Abstract

Sleep disruption in nursing home (NH) residents can lead to difficulties with activities of daily living, falls, depression, and decreases in memory, and mobility. Staff-delivered interventions are the most logical approach to improving NH resident sleep. But it can be difficult for frontline NH staff to implement quality improvement (QI) due to chronic understaffing, limited staff training, and challenges with data collection. We conducted a pilot multisite pragmatic clinical trial of the “LOCK” sleep program in three NHs. LOCK is an evidence-based framework using frontline huddling for QI uptake: “Learn from the bright spots” (local evidence of positive change), “Observe” (collect data through observation), “Collaborate in huddles” (conduct short frontline huddles), and “Keep it bite-size” (limit activities to 5-15 minutes). We used actiwatches to measure day and night activity and conducted 35 group interviews with leadership/champion implementation teams. We used an iterative, rapid-content analytic approach for qualitative analyses, examining factors associated with implementation variation using the Consolidated Framework for Implementation Research. In qualitative analyses, staff indicated the program was highly feasible and acceptable. From actiwatch data, for enrolled NH residents with ≥9 weeks of intervention exposure (n=11), total daytime activity increased an average of 19 minutes per day (SD=95) and total night-time sleep increased an average of 37 minutes per night (SD=39). The LOCK program is a promising approach and framework for NH staff pursuing QI related to sleep or other topics.

